# In Vitro Exploration of Dark Cytotoxicity of Anthocyanin-Curcumin Combination, A Herbal Photosensitizer

**DOI:** 10.7759/cureus.56714

**Published:** 2024-03-22

**Authors:** Dhanya M, Umamaheswari T.N, Rajalakshmanan Eswaramoorthy

**Affiliations:** 1 Department of Oral Medicine and Radiology, Saveetha Dental College and Hospitals, Saveetha Institute of Medical and Technical Sciences, Saveetha University, Chennai, IND; 2 Department of Biomaterials, Saveetha Dental College and Hospitals, Saveetha Institute of Medical and Technical Sciences, Saveetha University, Chennai, IND

**Keywords:** photosensitizer, oral mucosal lesions, photodynamic therapy, resource efficiency, innovation, natural resources, anthocyanin-curcumin

## Abstract

Background: Photodynamic therapy can be used to treat a variety of lesions noninvasively, including oral cancer. High-power laser therapy has also been used to treat oral squamous cell carcinomas. The two main components of photodynamic treatment are the photosensitizer and the light source. Herbal formulations of photosensitizers are used to mask the disadvantages of other photosensitizers.

Methodology: A methanol-diluted 25 grams of Punica granatum was used to create an anthocyanin extract using the flash evaporation method. Dimethyl sulfoxide (DMSO) was used as the first dilution agent for curcumin; later further dilution was done with distilled water. Following that, MCF-7 cells (a cancer cell line) were cultured with the produced samples, and the mono-tetrazolium salt (MTT) assay was used to determine the vitality of the cells.

Results: Cell reduction was significantly evident in all three groups, but the most significant cell death was found in the anthocyanin-curcumin group, at 29%.

Conclusion: The combination of anthocyanin-curcumin has one of the photophysical properties (dark cytotoxicity) and hence can aid as a photosensitizer.

## Introduction

Photodynamic therapy (PDT) is an upcoming new therapeutic strategy employed in a wide variety of oral lesions and oral malignancies [1]. Oral potentially malignant disorders are precursors of oral malignancies as it is associated with the use of tobacco [2]. The triad of photodynamic therapy involves the photosensitizer, a light source of appropriate wavelength and the reactive oxygen species production [3].

Photodynamic therapy works on the principle of non-thermal photochemical reactions [4]. The two main mechanisms of photodynamic therapy are apoptosis and necrosis. Necrosis was observed more frequently when using a higher dose of light source, whereas apoptosis was seen in lower doses of light source [5]. After the activation of a photosensitizer with a light source, the cytotoxic free radicals are released and subsequently destroy the targeted cells [6].

Photosensitizers play a vital role in photodynamic therapy with a high potential for tumor cells, thereby leading to better tumor response and decreased skin photosensitization [7]. Photosensitizers are derived solely from three classes, namely chlorophyll, porphyrins, and dyes [8]. Various conventional photosensitizers used in photodynamic therapy are methylene blue, 5-aminolevulinic acid, porphyrins, and erythrosine groups [9,10].

Various treatment strategies are carried out, which include conventional and herbal formulations [11]. Curcumin from the rhizome group with all the anti-inflammatory, anti-oxidant, and anti-microbial properties proves to possess bioactivity in the tumour cells when combined with a light source [12]. Anthocyanins are a class of polyphenols present in various fruits, flowers, and vegetables, and they naturally possess antioxidant properties that help in the production of reactive oxygen species. It is also evident that they can absorb light sources from 280-400 nanometres when excited [13]. The rationale behind this study was to explore the effectiveness of the herbal photosensitizer (anthocyanin + curcumin) by evaluating its cytotoxic properties in the dark. 

Aim and objectives

The study aims to assess the cytotoxicity of the herbal photosensitizer in the absence of light exposure. The main objective of the research was to determine the degree of cell viability in each group.

## Materials and methods

Study design and Study setting

This in vitro investigation was conducted at the Centre for Molecular Medicine and Diagnostics at Saveetha Institute of Medical and Technical Sciences. Because the study was conducted in vitro, G-power estimates were not conducted.

Sample groups

**Figure 1:**
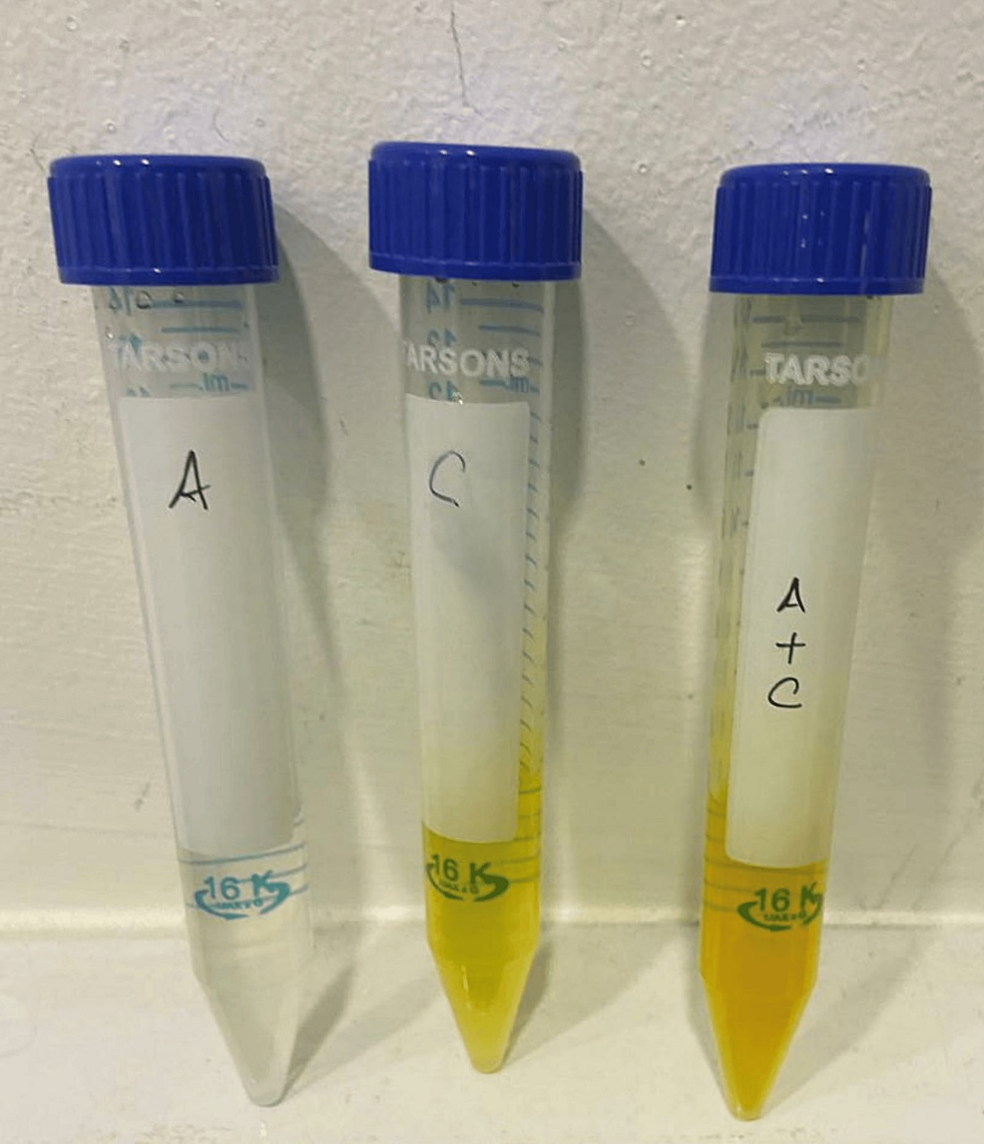
Sample groups Illustrates the three experimental groups obtained from their parent compounds. Sample A shows anthocyanin, Sample C shows curcumin and Sample A+C shows the combination of anthocyanin and curcumin.

Four sets of samples were used in this investigation. The cancer cell line MCF-7 was only present in the control group-Group One. 100 millilitres of water contained 0.5 microliters of anthocyanin in a diluted form in Group Two. Likewise, 100 millilitres of water contained 0.5 microliters of curcumin in Group Three. Group four consisted of 100 millilitres of water with 0.25 microliters of anthocyanin and 0.25 microliters of curcumin (Figure 1).

Preparation of curcumin and anthocyanin extract

**Figure 2:**
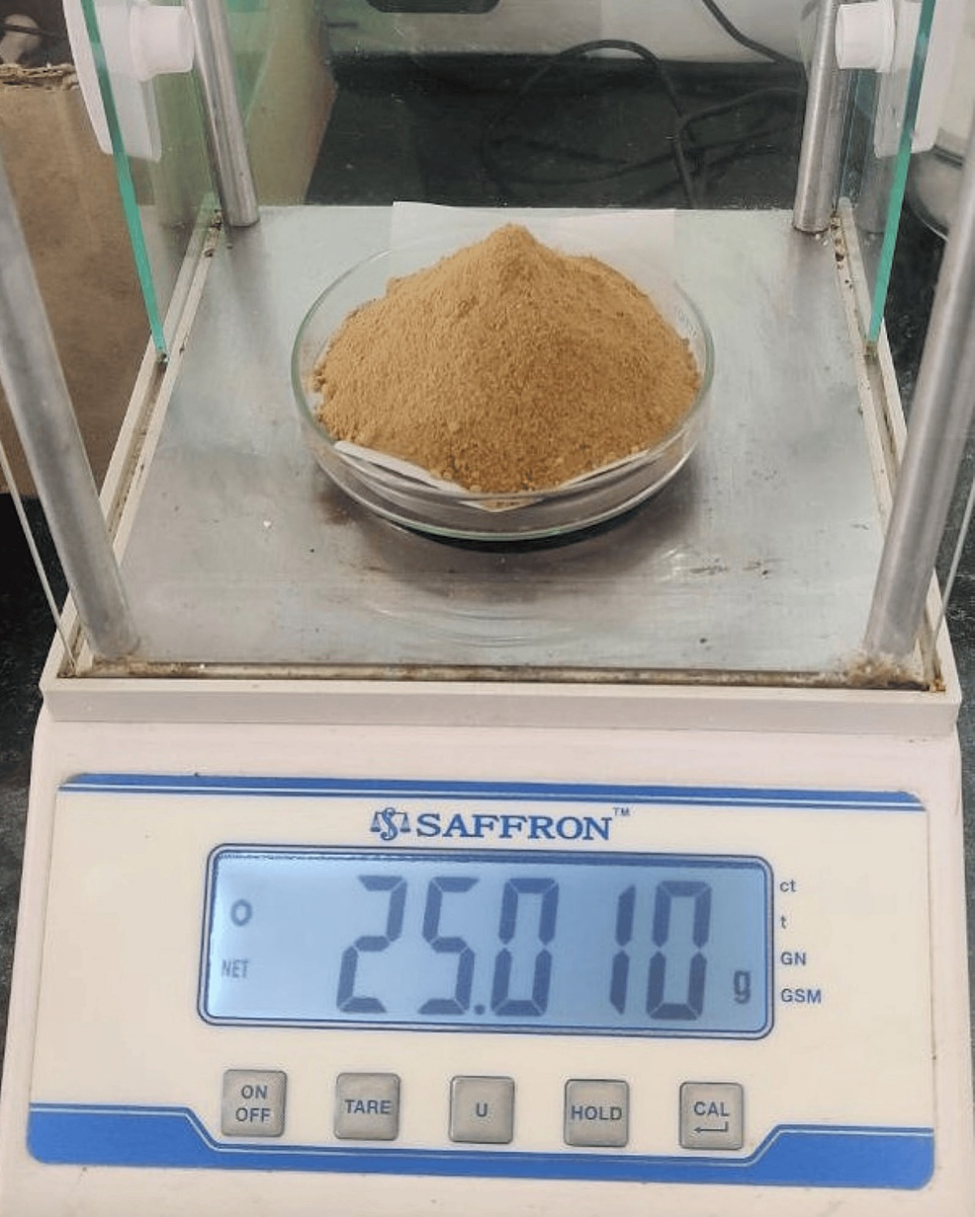
Peel powder of Punica granatum Parent anthocyanin compound that was isolated from 25 grams of Punica granatum.

**Figure 3:**
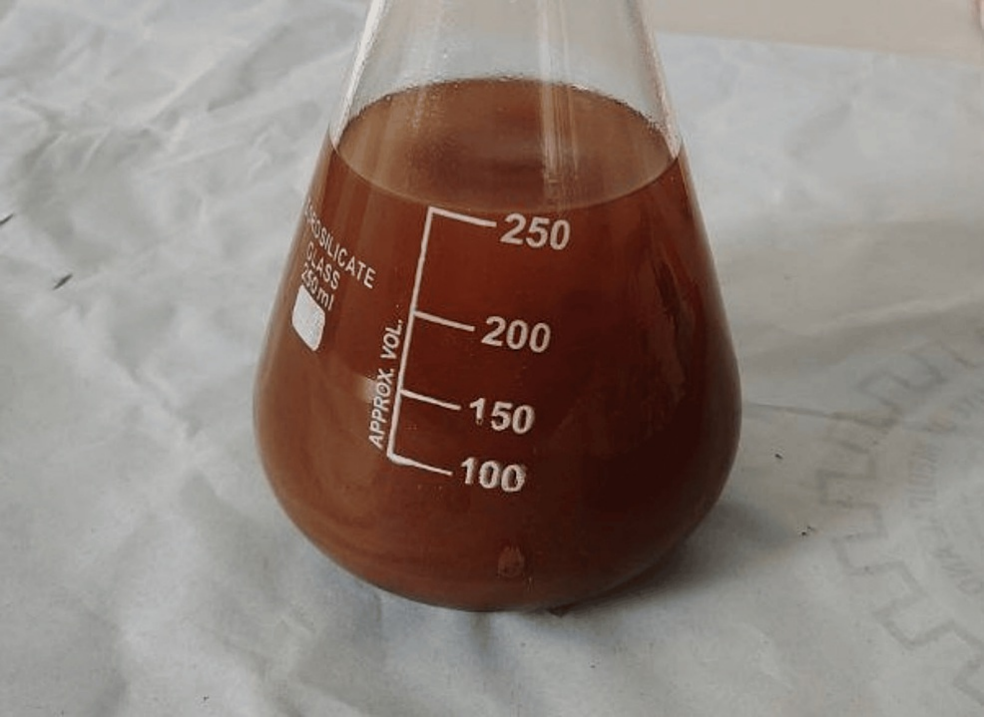
Dilution of Punica granatum peel powder Depicting the dissolved form of weighed Punica granatum peel powder in a conical flask containing methanol.

**Figure 4:**
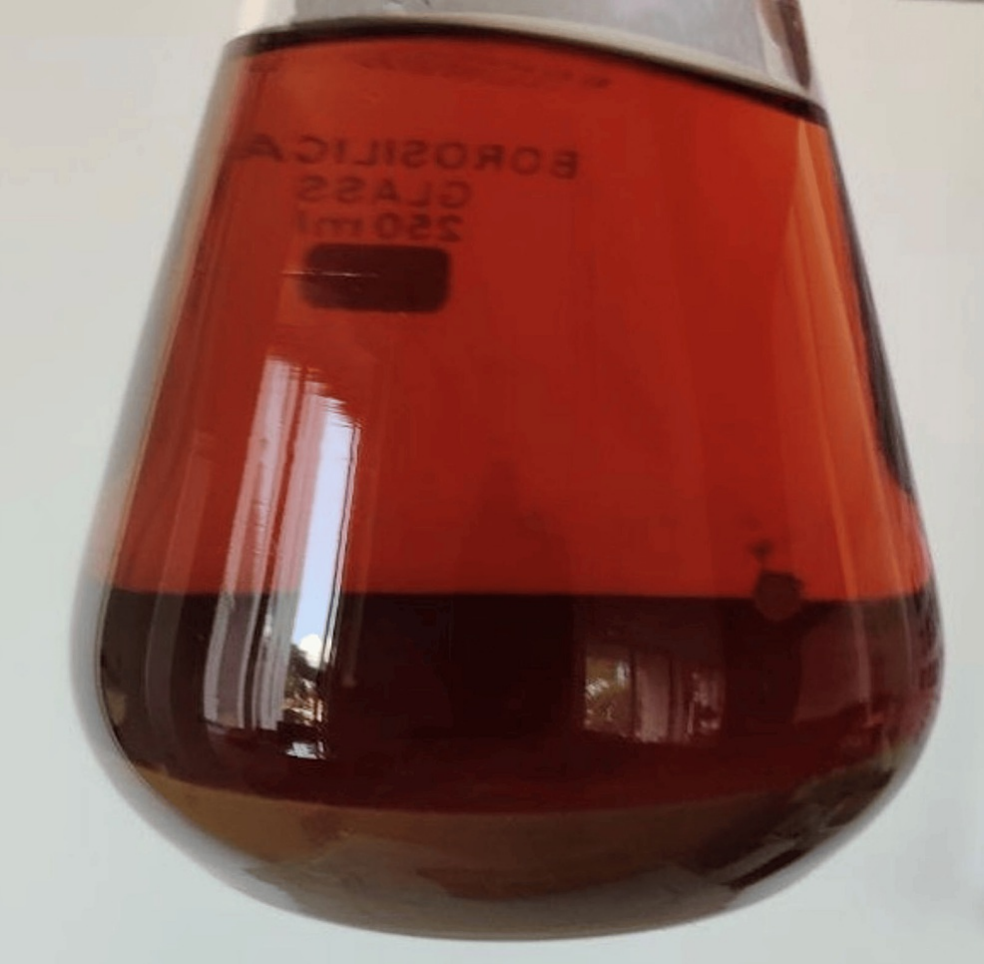
Post-trituration of anthocyanin compound Showing the dissolved Punica granatum compound in its sedimented state at the bottom of a 250 ml conical flask.

**Figure 5:**
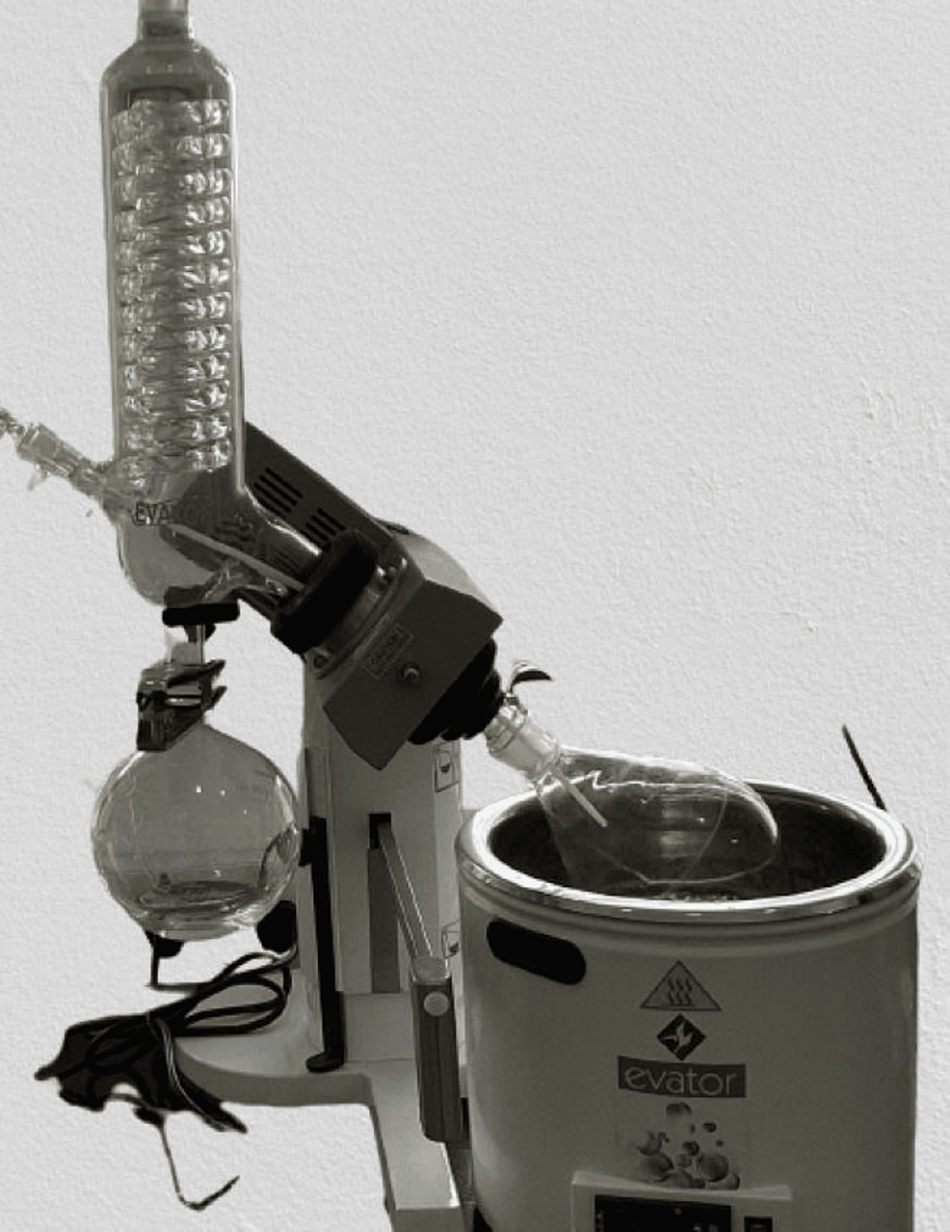
Flash evaporation of compound anthocyanin Illustrates the process of flash evaporation carried out at 32°C, which allows the compound to travel through several chambers and reach its purest state with continuous drop in temperature and pressure.

Molecular weight calculations of both the curcumin and anthocyanin were carried out prior to the sample preparation. Anthocyanin weighing 25 grams was extracted from the powdered peel of Punica granatum, which had been grated and weighed (Figure 2) and then it was diluted with 250 ml of methanol (Figure 3). The sedimented compound (Figure 4) was subjected to flash evaporation at 37°C (Figure 5). This flash evaporator had a rotation speed of 30-270 rpm with a temperature display, motorised heating bath controller, and feed stopcock tubes which were made up of PTFE (polytetraethylene).

**Figure 6:**
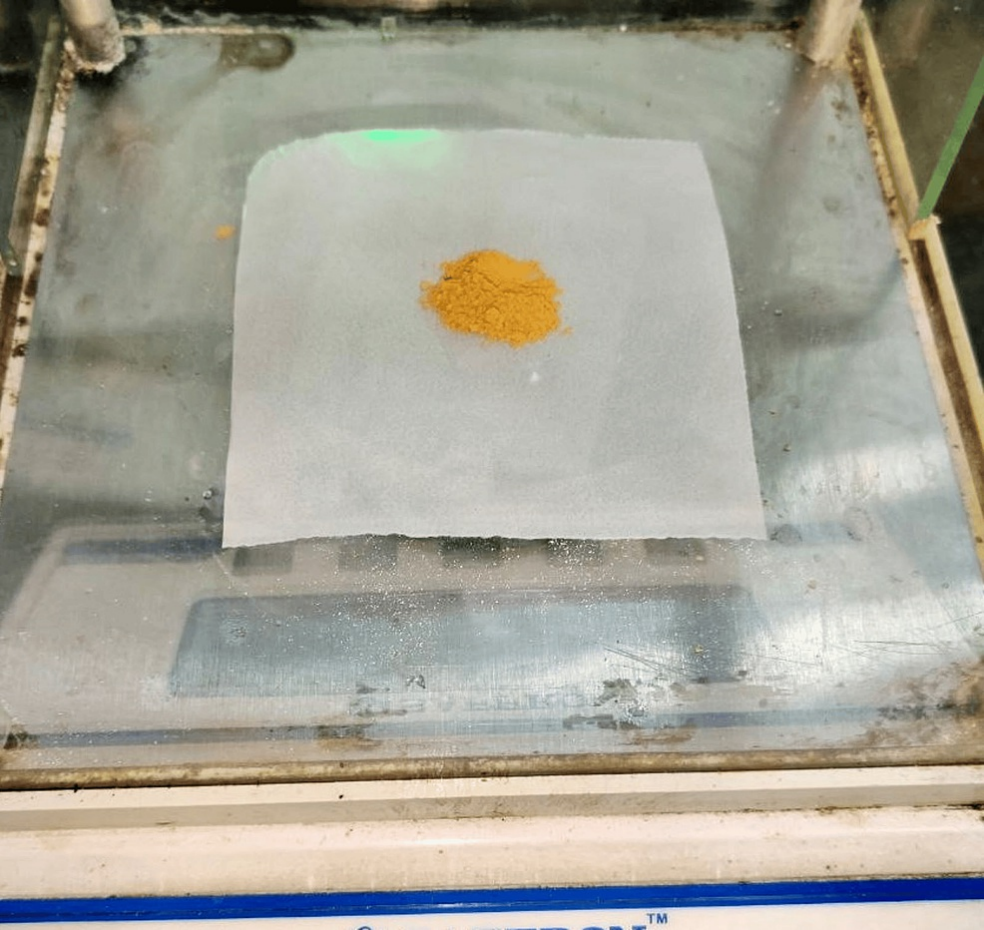
Curcumin extract from curcuma longa species Depicting the curcumin extract from Curcuma longa species weighed in an electronic weighing machine.

**Figure 7:**
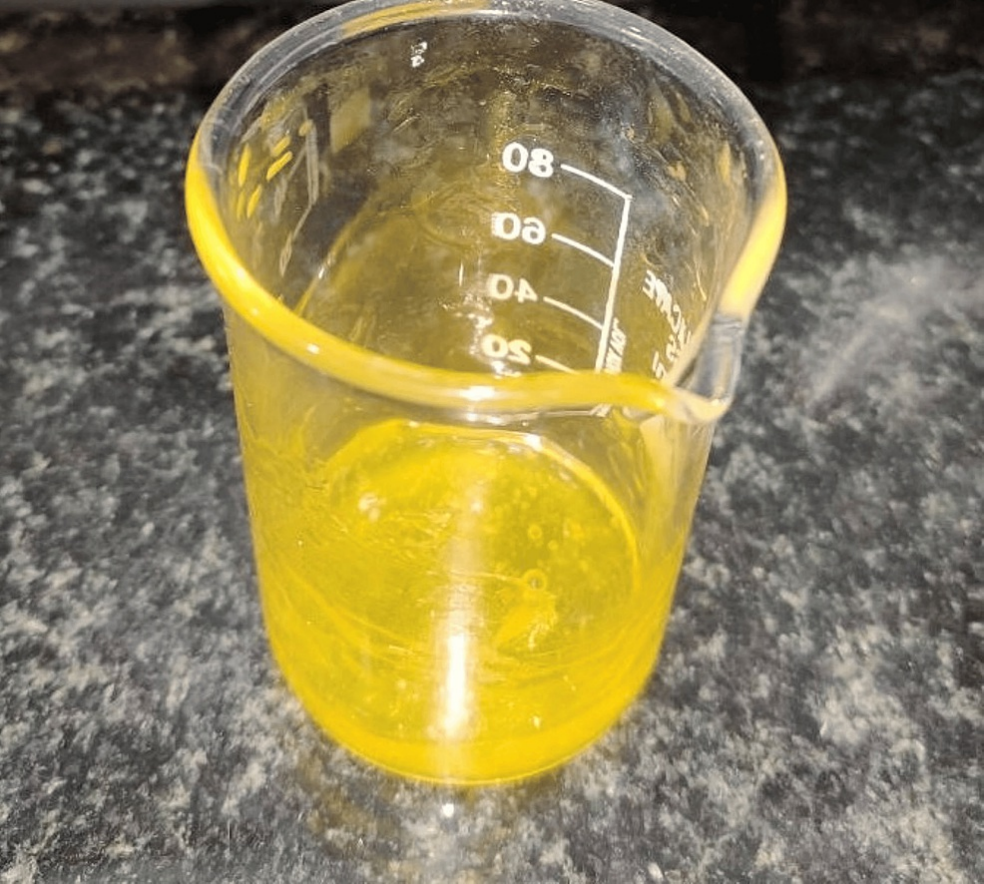
Initial dilution of curcumin Illustrates the initial dilution of curcumin extract from Curcuma longa species with two millilitres of dimethyl sulfoxide solution.

**Figure 8:**
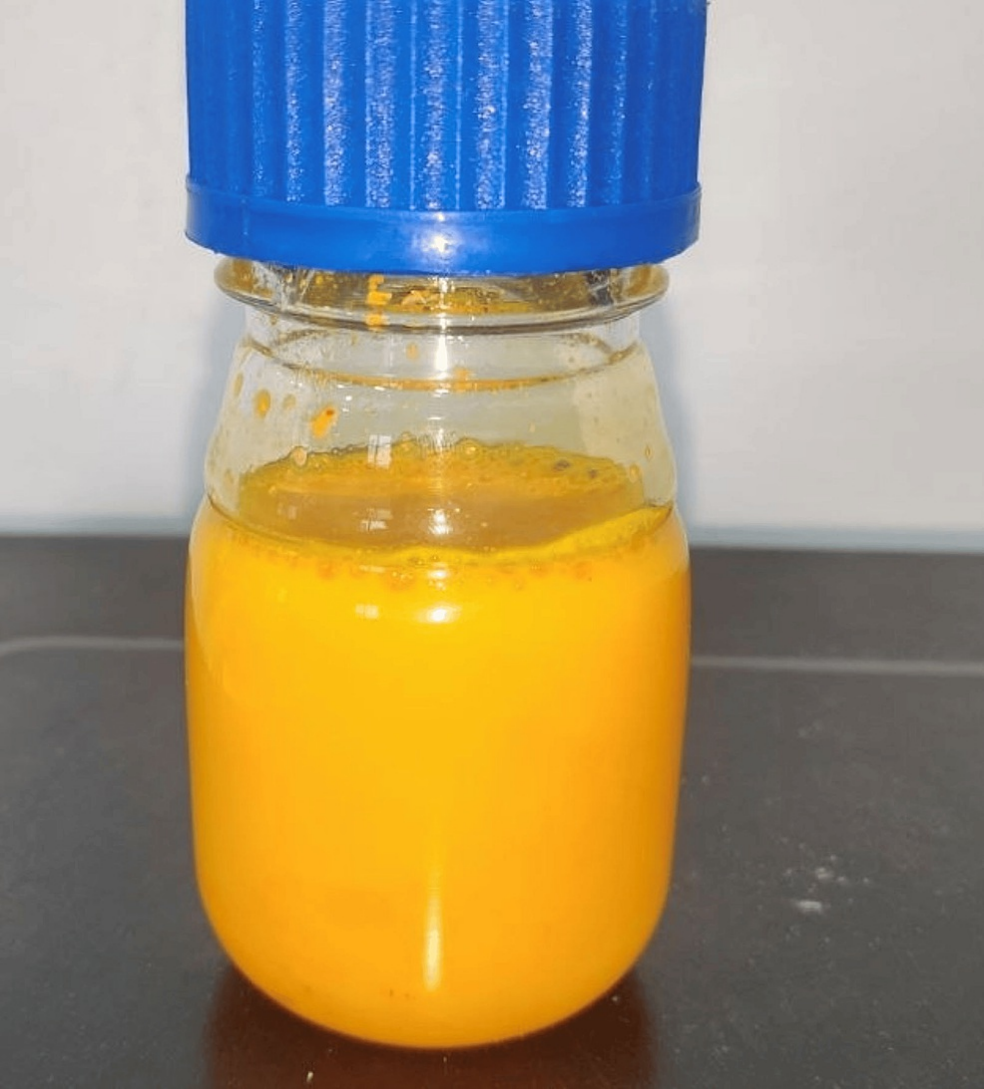
Secondary dilution of curcumin Depicting the secondary dilution of curcumin extract from Curcuma longa species with 100 ml of distilled water.

The 0.50 microliter sample of anthocyanin was further reduced to 100 millilitres of purified water from the extracted pure form. Curcumin was separated from its Curcuma longa species and weighed (Figure 6). Dilution was carried out twice with dimethyl sulfoxide (DMSO) (Figure 7) and 100 ml of distilled water (Figure 8).

Evaluation of cytotoxicity in dark

MCF-7 cells were cultivated in the absence of light in order to assess the cytotoxic activity in the dark with three different compounds. After the incubation period ended, the cells were washed, and the MTT (mono-tetrazolium salt) viability test was used to assess their viability. The MTT viability test was conducted using four primary mechanisms: the quantity of reagent penetrating the cells, the total quantity of cells present, their ingestional activity, and the time needed by the cells to generate formazan crystals. The cells were cultivated at room temperature and no specific instrumentation or model number was used. These cells were cultivated in a dark environment without any speck of light passing through it.

Statistical analysis 

Statistical analysis and error bars were calculated using SPSS software, version 23.0 (IBM Corp., Armonk, NY), and ANOVA tests were used to achieve these results.

## Results

**Figure 9:**
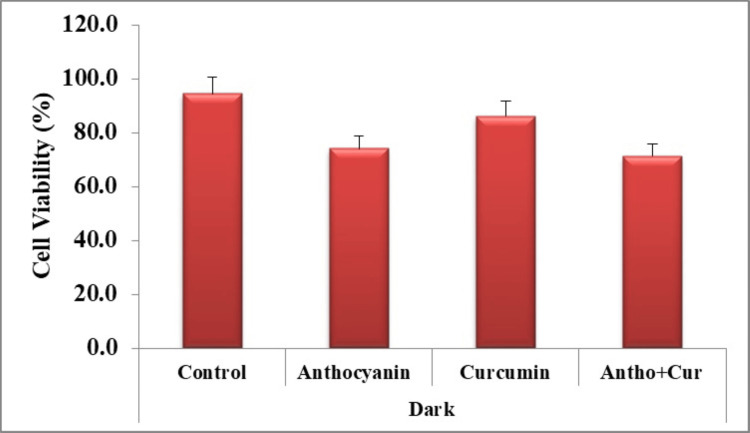
Cell survival rate amongst the control and the experimental groups

**Figure 10:**
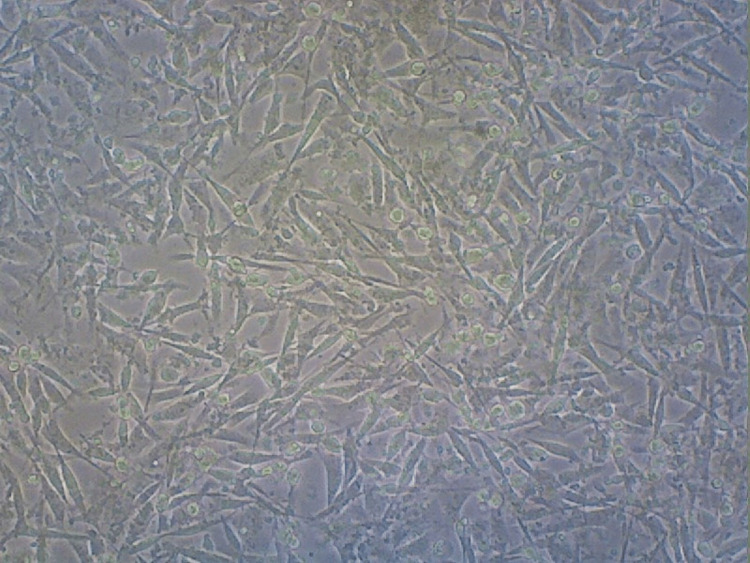
Cellular population in the control group This shows that the control group contains a higher cellular population of MCF-7 cells (cancer cell line).

**Figure 11:**
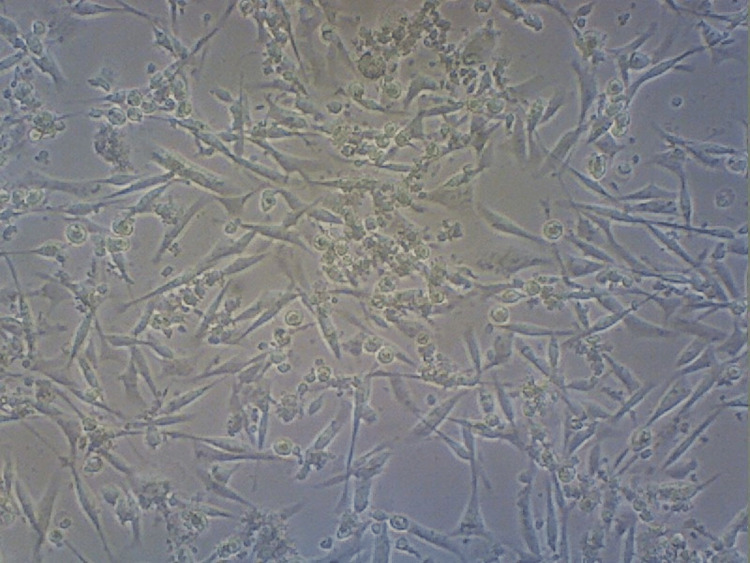
Cell survival rate in the anthocyanin group The cell rate of MCF-7 cells decreased by about 26% compared to that of the control group.

A graph was created to display the group's average cell viability percentage (Figure 9). According to the findings, 94.4% of the cells in the control group demonstrated viability (Figure 10). A cellular reduction of 26% was observed in the anthocyanin group compared to the controls (Figure 11).

**Figure 12:**
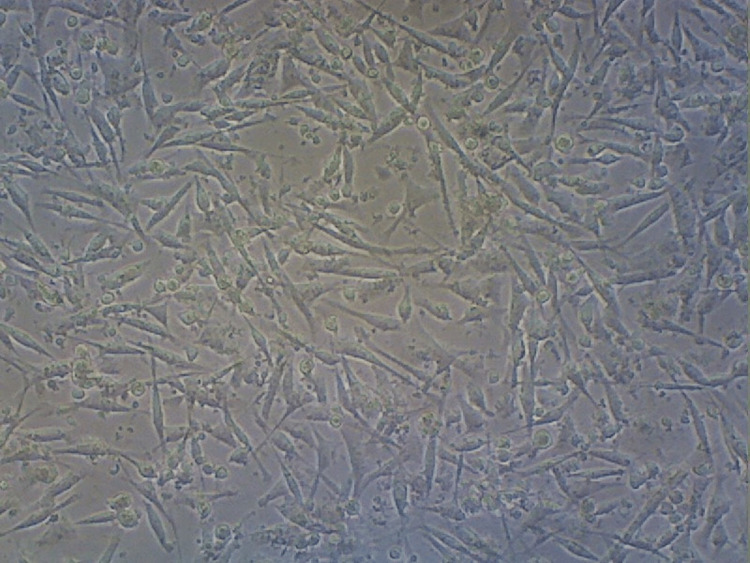
Cell survival rate in the curcumin group Showing a reduction in cell rate of about 14% compared to the control group.

**Figure 13:**
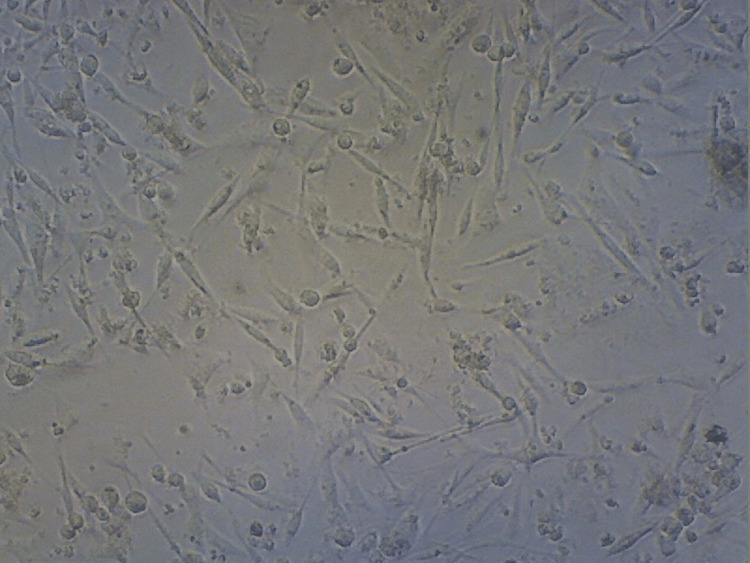
Cell survival rate in the curcumin + anthocyanin group This illustrates a significant decrease in the cellular rate by 29%, which was found to be superior to all the other groups.

The curcumin group showed 14% of cell death (Figure 12). Similarly, the group containing the combination of the compounds demonstrated a significantly higher cellular death rate of about 29% compared to that of other sample groups (Figure 13).

## Discussion

Herbal preparation of photosensitizer allows for masking up the limitations of conventional photosensitizers, such as high grades of burning sensation, pain, pruritus, oedema, and local irritation [14,15]. Thus in this current study curcumin and anthocyanin compounds are used as experimental groups. In a study by Kazantzis et al., the dark cytotoxic photo property of curcumin was evaluated using LNcaP cells, and the results were in correlation with the current study, which proved the presence of dark cytotoxicity. In this current study, the experimental groups were assessed for dark cytotoxic properties by incubating them with MCF-7 cells [16].

A study by Leite et al. used curcumin as a mouth rinse combined with a blue light source and evaluated its efficacy; they used a stock solution of curcumin at a concentration of 30 mg/litre [17]. In this current study post assessing the molecular weights of the single compounds, concentrations of 0.5 microliter of curcumin, 0.5 microliter of anthocyanin, and 0.25 + 0.25 microliter of combination of anthocyanin and curcumin were used. The cytotoxicity of herbal gels comprising tulsi, aloe vera, and turmeric was investigated previously by Dhanvanth et al. using an Enzyme-Linked Immunosorbent Assay (ELISA) [18]. We assessed dark cytotoxicity in the present investigation using the MTT test.

Photodynamic therapy has been utilised in studies by Chen et al. [19] and Cosgarea et al. [20] for oral leukoplakia, oral verrucous hyperplasia, and oral lichen planus, with conventional 5-aminolevulinic acid as a photosensitizer. Similarly, studies by Aghahosseini et al. [21] and Mirza et al. [22] assessed the efficacy of photodynamic therapy in oral lichen planus using methylene blue as a photosensitizer, which proved a significant reduction in the lesion size by 44.3%. In this current study, the prepared herbal photosensitizer will aid in biocompatibility and will be used in clinical trials of various oral mucosal lesions in the future.

Since this was a novel preparation, the study focused on exploring whether this herbal preparation had the potential to function as a photosensitizer by assessing certain photophysical properties. No special instruments were used in the study except the flash evaporator (specifications mentioned in the methodology section); furthermore, this study was conducted with the available natural herbal compounds that were assessed with MTT Assay. 

Limitations

In addition to a number of therapeutic benefits, the current study fell short of several requirements, in that it did not compare the results with photosensitizers that are often employed, like 5-aminolevulinic acid and toluidine blue. 

Future scopes 

Future studies could focus on the evaluation of the presence of other photophysical properties, such as reactive oxygen species production and UV absorption spectra. After assessing the photophysical properties, this herbal photosensitizer could be used in clinical trials, hence helping alleviate the progression of malignant transformation. Further future studies will aid in comparing the effectiveness of this herbal photosensitizer with that of conventional photosensitizers such as methylene blue and 5-ALA (aminolevulinic acid) in a laboratory setup followed by clinical trials. This herbal preparation aids in various clinical implications by masking up the adverse reactions of the conventional photosensitizers and can also act as a conservative therapy in minor oral lesions where conventional therapies are contraindicated.

## Conclusions

In conclusion, the study aids as a novel therapeutic approach in the formulation of herbal photosensitizer with minimal cumulative toxicity. From this study, it was evident that the combination of anthocyanin and curcumin proved to have a higher cellular reduction rate and hence could be used as a photosensitizer in photodynamic therapy.

## References

[1] Le MN, Wuertz BR, Biel MA, Thompson RL, Ondrey FG (2022). Effects of methylene blue photodynamic therapy on oral carcinoma and leukoplakia cells. Laryngoscope Investig Otolaryngol.

[2] Shilpa S, Uma Maheswari TN (2020). Prevalence, forms and types of tobacco smoking- a literature. J Biology Pharm Allied Sci.

[3] Peng W, de Bruijn HS, Ten Hagen TL (2020). In-vivo optical monitoring of the efficacy of epidermal growth factor receptor targeted photodynamic therapy- the effect of fluence rate. Cancers (Basel).

[4] Kübler AC (2005). Photodynamic therapy. J Medical Laser Application.

[5] Jajarm HH, Falaki F, Sanatkhani M, Ahmadzadeh M, Ahrari F, Shafaee H (2015). A comparative study of toluidine blue-mediated photodynamic therapy versus topical corticosteroids in the treatment of erosive-atrophic oral lichen planus: a randomized clinical controlled trial. Lasers Med Sci.

[6] Nagi R, Muthukrishnan A, Rakesh N (2023). Effectiveness of photodynamic therapy (PDT) in the management of symptomatic oral lichen planus -a systematic review. J Oral Biol Craniofac Res.

[7] Garg AD, Bose M, Ahmed MI, Bonass WA, Wood SR (2012). In vitro studies on erythrosine-based photodynamic therapy of malignant and pre-malignant oral epithelial cells. PLoS One.

[8] Allison RR, Downie GH, Cuenca R, Hu XH, Childs C, Sibata CH (2004). Photosensitizers in clinical PDT. J Photodiagnosis Photodyn Ther.

[9] Kofler B, Romani A, Pritz C, Steinbichler TB, Schartinger VH, Riechelmann H, Dudas J (2018). Photodynamic effect of methylene blue and low level laser radiation in head and neck squamous cell carcinoma cell lines. Int J Mol Sci.

[10] Choudhary R, Reddy SS, Nagi R, Nagaraju R, Kunjumon SP, Sen R (2022). The effect of photodynamic therapy on oral-premalignant lesions- a systematic review. J Clin Exp Dent.

[11] Dhanvanth M, Uma Maheswari TN (2022). Topical herbal therapeutic formulation used in the management of oral potentially malignant disorders- a systematic review. J Indian Academy of Oral Med Radio.

[12] Ailioaie LM, Ailioaie C, Litscher G (2021). Latest Innovations and nanotechnologies with curcumin as a nature-inspired photosensitizer applied in the photodynamic therapy of cancer. Pharmaceutics.

[13] Oliveira H, Correia P, Pereira AR (2020). Exploring the applications of the photoprotective properties of anthocyanins in biological systems. Int J Mol Sci.

[14] Mostafa D, Moussa E, Alnouaem M (2017). Evaluation of photodynamic therapy in treatment of oral erosive lichen planus in comparison with topically applied corticosteroids. Photodiagnosis Photodyn Ther.

[15] Wong SJ, Campbell B, Massey B (2013). A phase I trial of aminolevulinic acid-photodynamic therapy for treatment of oral leukoplakia. Oral Oncol.

[16] Kazantzis KT, Koutsonikoli K, Mavroidi B (2020). Curcumin derivatives as photosensitizers in photodynamic therapy: photophysical properties and in vitro studies with prostate cancer cells. Photochem Photobiol Sci.

[17] Leite DP, Paolillo FR, Parmesano TN, Fontana CR, Bagnato VS (2014). Effects of photodynamic therapy with blue light and curcumin as mouth rinse for oral disinfection: a randomized controlled trial. Photomed Laser Surg.

[18] Dhanvanth M, Uma Maheswari TN, Rajeshkumar S (2020). Anti-inflammatory effect of herbal formulation of tulsi, aloe vera and turmeric aqueous extract. Int J Pharm Res.

[19] Chen HM, Yu CH, Tsai T, Hsu YC, Kuo RC, Chiang CP (2007). Topical 5-aminolevulinic acid-mediated photodynamic therapy for oral verrucous hyperplasia, oral leukoplakia and oral erythroleukoplakia. Photodiagnosis Photodyn Ther.

[20] Cosgarea R, Pollmann R, Sharif J (2020). Photodynamic therapy in oral lichen planus: a prospective case-controlled pilot study. Sci Rep.

[21] Aghahosseini F, Arbabi-Kalati F, Fashtami LA, Djavid GE, Fateh M, Beitollahi JM (2006). Methylene blue-mediated photodynamic therapy: a possible alternative treatment for oral lichen planus. Lasers Surg Med.

[22] Mirza S, Rehman N, Alrahlah A, Alamri WR, Vohra F (2018). Efficacy of photodynamic therapy or low level laser therapy against steroid therapy in the treatment of erosive-atrophic oral lichen planus. Photodiagnosis Photodyn Ther.

